# Associations of estimated glucose disposal rate with chest pain prevalence and mortality risks: a population-based study

**DOI:** 10.1186/s12872-026-06336-0

**Published:** 2026-07-25

**Authors:** Jieke Wang, Manli Ye

**Affiliations:** 1 Department of Hand and Plastic Surgery, The Second Affiliated Hospital & Yuying Children’s Hospital of Wenzhou Medical University, Wenzhou, Zhejiang 325000 China; 2 Clinical Laboratory Center, The Second Affiliated Hospital & Yuying Children’s Hospital of Wenzhou Medical University, NO.1111, East Section of Wenzhou Avenue, Longwan District, Wenzhou City, Zhejiang Province 325000 China

**Keywords:** eGDR, Insulin resistance, All-cause mortality, Risk stratification

## Abstract

**Background:**

Estimated glucose disposal rate (eGDR), a validated surrogate for insulin resistance, is associated with cardiovascular disease, but its relationship with chest pain, which is a common presenting symptom of cardiovascular events, remains unclear. This study investigated the associations of eGDR with chest pain prevalence and all-cause mortality.

**Methods:**

Using cross-sectional and cohort study designs, we analyzed data from 1999 to 2018 National Health and Nutrition Examination Survey (NHANES). Survey-weighted logistic regression was used to examine the association between eGDR and chest pain prevalence, while weighted Cox model was used to evaluate the relationship between eGDR and all-cause mortality in participants with and without chest pain. Restricted cubic splines (RCS) examined potential nonlinearity, and Kaplan–Meier curves visualized survival differences by eGDR category. Mediation analysis examined whether white blood cells (WBC) count mediated the eGDR–mortality association.

**Results:**

The study included 13,161 participants, with the sample being weighted to reflect a population of 54,616,985 individuals. After full adjustment, each standard deviation (SD) increase in eGDR correlated with an 11% lower odds of chest pain (*P* < 0.001). Cox regression revealed each SD rise in eGDR was associated with a 7% lower risk of all-cause mortality among participants with chest pain (*P* = 0.004). RCS confirmed linear relationships for eGDR with both chest pain prevalence and mortality risk, irrespective of chest pain status. When eGDR was dichotomized at the population median (7.34 mg/kg/min), participants with high eGDR was significantly associated with reduced odds of chest pain (OR = 0.67, 95% CI: 0.59–0.77, *P* < 0.001) and lower all-cause mortality (HR = 0.85, 95% CI: 0.75–0.95, *P* = 0.006) compared to those with low eGDR. Kaplan–Meier curves demonstrated a clear survival advantage for participants with higher eGDR. WBC mediated 20.04% of the association between eGDR and all-cause mortality (*P*_indirect_ < 0.001).

**Conclusion:**

Higher eGDR is robustly associated with lower chest pain prevalence and reduced all-cause mortality risk, irrespective of chest pain status. The partial mediation by WBC suggests a potential inflammatory pathway. These findings support the potential value of eGDR in non-invasive cardiometabolic risk stratification.

**Supplementary Information:**

The online version contains supplementary material available at https://doi.org/10.1186/s12872-026-06336-0.

## Background

Within the United States of America, chest pain ranks as the second most common reason for emergency department visits after injury, accounting for roughly 6.5–7.8 million visits annually [1, 2]. Up to 40% of patients revisit the emergency department for chest pain within a year [3]. The economic burden of acute chest pain is substantial, with emergency visit costs alone estimated at $13-$15 billion [4, 5]. Chest pain can arise from a wide spectrum of conditions, ranging from benign to life-threatening [6–8]. Its differential diagnosis includes acute aortic syndrome, acute coronary syndrome, myocarditis, pericarditis, pulmonary embolism, peptic ulcer, pneumonia, and pneumothorax [2]. Cardiovascular disease (CVD), particularly coronary artery disease, a common underlying cause of chest pain, affects over 18.2 million adults in the U.S. and remains the leading cause of death for both genders [9–11]. Identifying factors associated with chest pain is therefore crucial.

Insulin resistance (IR), characterized by reduced insulin-mediated glucose uptake, is an established risk factor for both microvascular and macrovascular complications [12].

Regarded as the gold standard, the hyperinsulinemic-euglycemic (HIEG) clamp offers the most accurate assessment of insulin sensitivity [13]. However, its invasive characteristics and high expense may limit its use in clinical settings. Common alternatives for screening IR comprise the homeostasis model assessment of insulin resistance (HOMA-IR) and the estimated glucose disposal rate (eGDR) [14, 15]. HOMA-IR relies on fasting insulin and glucose levels, and its accuracy can be compromised by exogenous insulin administration, particularly in patients with diabetes [14]. In contrast, eGDR is based on measurements of waist circumference, hemoglobin A1c (HbA1c), and hypertension status, marking it an accessible, reliable, and economical IR marker. Studies from the past have found that a lower eGDR, reflecting greater insulin resistance, is connected with an elevated risk of cardiovascular incidents, including ischemic stroke, atrial fibrillation, myocardial infarction, heart failure (HF) and arterial stiffness [16–19], and that eGDR holds prognostic value for cardiovascular outcomes [20, 21]. To our knowledge, however, limited existing literature has investigated the association between eGDR and chest pain, which is the most prevalent concomitant symptom linked to cardiovascular events.

Emerging evidence indicates that the clinical implications of eGDR extend beyond cardiovascular events to mortality outcomes [22]. A population-based cohort study has demonstrated that a lower eGDR is independently associated with an increased risk of all-cause mortality in both diabetic and non-diabetic populations, and that this association persists after extensive multivariable adjustment [23]. The mechanisms linking lower eGDR to increased mortality are likely multifactorial, involving accelerated atherosclerosis, endothelial dysfunction, systemic inflammation, and an elevated burden of cardiometabolic comorbidities—pathways that are particularly active in patients presenting with chest pain [18, 24, 25]. Accordingly, among individuals evaluated for chest pain, those with lower eGDR may represent a subgroup at disproportionately high risk of future fatal events. However, dedicated studies examining the prognostic value of eGDR for mortality specifically in chest pain populations remain scarce, and the extent to which eGDR can inform risk stratification in this common clinical scenario has not been comprehensively evaluated.

The present study aimed to investigate the associations of eGDR with chest pain prevalence and all-cause mortality in adults with and without chest pain, and to explore the potential mediating role of white blood cell (WBC) count in the eGDR–mortality relationship.

## Methods

### Study design and participants

The NHANES program undertakes a detailed and complex approach to the selection of a representative sample of the U.S. population biennially. Its primary purpose is to analyze the nutritional and health status of U.S. residents. To produce estimates that accurately reflect the demographic profile of the United States, appropriate sample weights, stratification, and clustering must be incorporated into all statistical analyses following CDC guidelines. For thorough details regarding the design of the survey sample and the methods used for weight calculation, visit https://wwwn.cdc.gov/nchs/nhanes/analyticguidelines.aspx. To maintain ethical standards, the survey received approval from the Institutional Review Board set up by the National Center for Health Statistics Institutional Review Board. Moreover, everyone who chose to take part voluntarily gave their written consent before being included in the study. The NHANES gathers various types of data, such as demographic details, questionnaire responses, laboratory findings, and medical examination results.

**Fig. 1:**
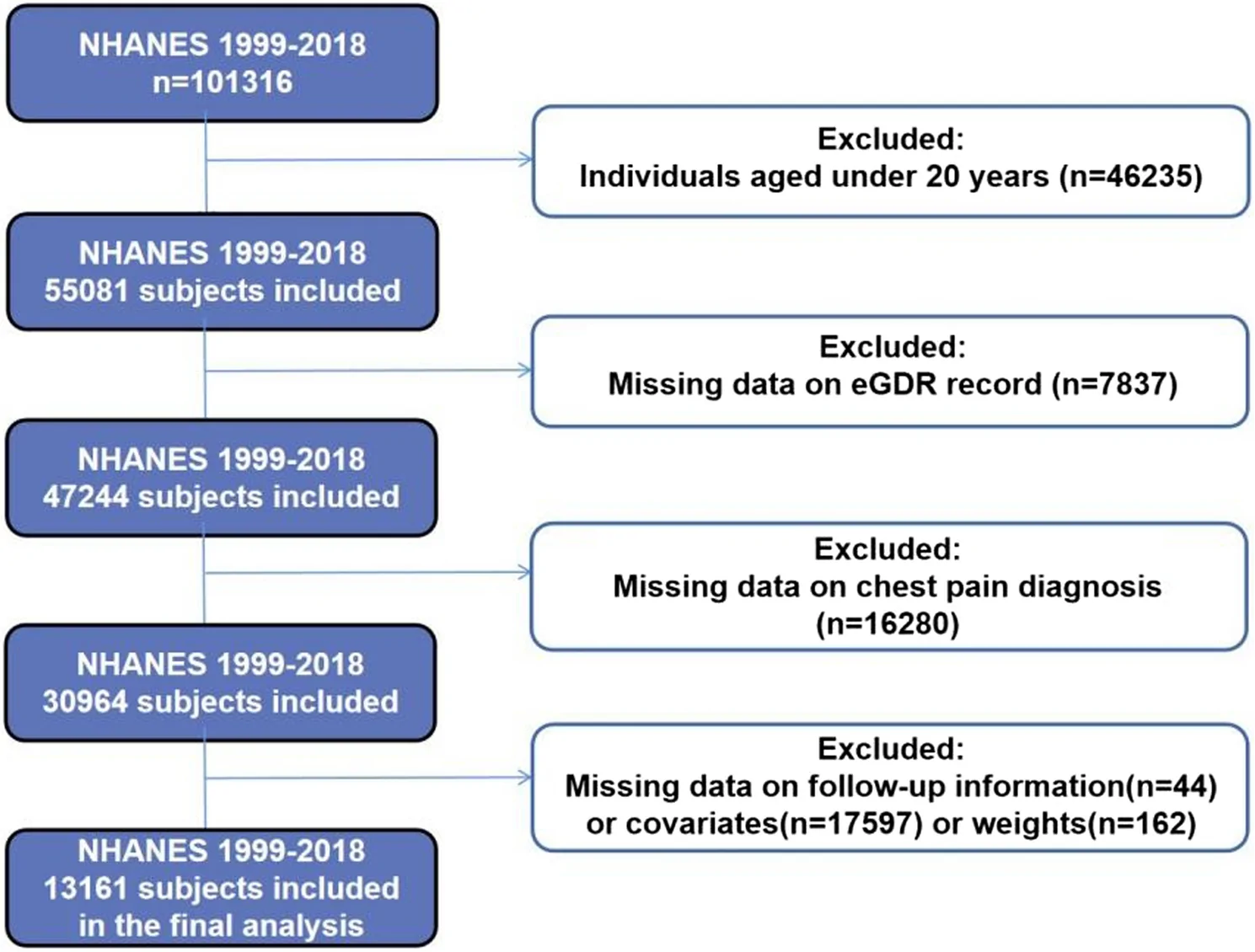
Flow chart for participants selection in NHANES

The NHANES 1999–2018 cycle included a sum of 101,316 participants. The research analysis utilized the remaining sample after excluding subjects lacking chest pain diagnosis information, eGDR records, covariates records, sample weights, and follow-up records (Fig. 1).

### Variable harmonization across survey cycles

To ensure comparability across survey cycles, all variables were harmonized following a standardized protocol. Specifically, we reviewed the variable names, coding schemes, and response categories for each variable across all cycles using the NHANES codebooks provided by the CDC. Variables that were collected with identical question wording and response options across cycles were directly merged without recoding. For variables with inconsistent coding across cycles, we recoded raw responses into uniform categories according to pre-specified harmonization rules. Categorical variables were harmonized by collapsing subcategories into consistent broader groups when necessary.

Continuous variables were kept on their original scales unless laboratory methods changed across cycles. All harmonization decisions were documented in a data dictionary prior to analysis, and the harmonized dataset was cross-validated against the original cycle-specific files to ensure accuracy.

### Exposure variable

eGDR was determined as the exposure variable using the equation: 21.158 - (0.09 × *waist circumference* [cm]) - (3.407 × *hypertension status* (1 = yes, 0 = no)) - (0.551 × *HbA1c* in %), acting as an indicator of IR.

### Outcome variables

The main outcomes assessed were the status of chest pain and mortality. A diagnosis of chest pain was made if the subject responded “yes” to the question “{Have you/Has SP} ever had any pain or discomfort in {your/her/his} chest?”. All-cause mortality was defined as death from any cause. Participant follow-up and endpoint information were gathered by aligning their records with the National Death Index until December 31st, 2019.

Participants alive at the end of follow-up (December 31st, 2019) were administratively censored. The ‘MORTSTAT’ variable was utilized to indicate death status, while ‘PERMTH_EXM’ was used for follow-up duration. Median follow-up time was estimated using the reverse Kaplan-Meier method.

### Sampling weight processing

All variables to calculate eGDR (waist circumference, mobile examination centers (MEC)-measured blood pressure, laboratory HbA1c) were collected during MEC examinations. Following National Center for Health Statistics (NCHS) analytic guidelines, MEC sampling weights were applied for all statistical models. Cycles 1999–2002 adopted the 4-year MEC weight WTMEC4YR, while 2003–2018 cycles used 2-year WTMEC2YR. We constructed unified pooled weights across all 10 survey cycles: (1) 1999–2002 participants: pooled_weight = WTMEC4YR × 2 / 10; (2) 2003–2018 participants: pooled_weight = WTMEC2YR × 1 / 10. Masked variance strata (SDMVSTRA) and pseudo-primary sampling units (SDMVPSU) were incorporated into all weighted models for cluster sampling error [26].

### Assessment of covariates

Covariates were chosen a priori based on literature review and biological plausibility.

Participants provided sociodemographic and lifestyle details through standardized questionnaires, which included questions on gender (female, male), age, race (Black, White, Mexican American, and Other), family poverty-to-income ratio (PIR), and educational attainment (below high school, high school or equivalent, and above high school). Body mass index (BMI) was calculated using weight (kg)/height (m^2^) [27].

Laboratory measurements, including WBC, total cholesterol (TC), fasting glucose, and HbA1c, were determined by standardized laboratory operating procedures. 

The definition of CVD status relied on self-reported diagnoses of HF, coronary heart disease (CHD), stroke, angina, or heart attack, which were verified by a medical physician [28]. The presence of hypertension was determined by the fulfillment of any of the subsequent criteria: (1) the individual’s systolic blood pressure was equal to or exceeded 140 mmHg, or their diastolic blood pressure was equal to or exceeded 90 mmHg; (2) the participant confirmed with a “yes” to the inquiry: “{Have you/Has SP} ever been told by a doctor or other health professional that {you/s/he} had hypertension, also called high blood pressure?”; (3) the participant was currently under treatment with antihypertensive medication [29]. Individuals were considered to have diabetes mellitus when they satisfied any of these conditions: had (1) a fasting plasma glucose level ≥ 7.0 mmol/L, (2) a HbA1c concentration ≥ 6.5%, (3) a diabetes diagnosis, or (4) self-reported usage of antidiabetic medications [30].

### Statistical analysis

The Kolmogorov–Smirnov test was employed to assess the normality of continuous data.

Variables that followed a normal distribution were presented as means with standard deviations and compared using the *t*-test. Data that deviated from normality were reported as medians with interquartile ranges (25th to 75th percentile) and compared using the Wilcoxon rank-sum test. Categorical variables were expressed as unweighted counts (weighted proportions) and compared using the Rao–Scott χ² test for survey data. All descriptive and baseline comparisons accounted for the complex survey design using appropriate sample weights, strata, and primary sampling units, and were performed with the survey package.

Weighted univariate and multivariate logistic regression analyses, performed with the survey package, were used to estimate the relationship between eGDR and chest pain, with findings expressed as odds ratios (ORs) and 95% confidence intervals (CIs).

Weighted univariate and multivariate Cox proportional hazard models were conducted using the survey package to estimate the association between eGDR and all-cause mortality in participants with or without chest pain, presented as hazard ratios (HRs) with 95% CIs. The proportional hazards assumption was tested using Schoenfeld residuals.

Kaplan–Meier curves were generated using the survival package to visualize survival differences across eGDR groups, and comparisons were conducted via the log-rank test.

In order to control confounding variables, three weighted logistic and Cox regression models were utilized: Model 1, no adjustments were made for covariates; Model 2, adjusted for gender, age, and race; and Model 3, adjusted for gender, age, race, education levels, BMI, TC, WBC, PIR, HF, CHD, heart attack, angina, diabetes mellitus, and stroke. Our models include covariates, which are variables picked due to their proven relationships with outcomes, as confirmed by extant scientific evidence.

To further assess potential linear or nonlinear relationships, restricted cubic spline (RCS) curves were generated using the rms package to examine the connections between eGDR and chest pain, along with eGDR and all-cause mortality. All RCS analyses were based on the fully adjusted model 3.

To provide a clinically interpretable categorical analysis while avoiding the coarseness of simple quartile grouping, eGDR was also dichotomized. In the absence of a clear threshold in the dose–response curve, the population median was prespecified as the cut-point. eGDR values below the median were classified as low eGDR and those at or above the median as high eGDR. This binary classification was used in both logistic regression models for chest pain and Cox regression models for mortality outcomes.

Stratified analyses were performed to evaluate the link between eGDR, the prevalence of chest pain, and mortality among prespecified subgroups, such as age (< 60, ≥ 60), gender, BMI (< 25, 25-<30, ≥ 30), PIR, race, and education level. Effect modification was assessed by including multiplicative interaction terms between eGDR and each subgroup variable in the fully adjusted models. Interaction *p*-values were reported. Given that the primary aim was to examine the consistency of the associations across subgroups rather than to test multiple independent hypotheses, no formal multiplicity adjustment was applied.

Subgroup analyses were exploratory, and the risk of false-positive findings should be considered when interpreting subgroup results. These analyses were performed using the survey and survival packages.

Mediation analyses were performed to evaluate whether systemic inflammation mediates the association between eGDR and all-cause mortality. We employed a counterfactual-based mediation framework, fitting a linear regression model for WBC (mediator) and a Cox proportional hazards model for all-cause mortality (outcome), both adjusted for Model 3 covariates. Because WBC serves as the mediator in this analysis, it was removed from the list of Model 3 covariates to avoid blocking the mediation pathway.

The indirect effect, direct effect, total effect, and proportion mediated were estimated using the product-of-coefficients method with 1,000 nonparametric bootstrap resamples to obtain 95% confidence intervals. All mediation analyses were performed using the mediation package in R.

Several sensitivity analyses were conducted to assess the robustness of the primary findings. First, we excluded participants with baseline self-reported cardiovascular disease. Second, we repeated the main analyses after excluding participants who died within the first 2 years of follow-up to reduce potential reverse causation. Third, we restricted the analysis to participants with complete smoking and alcohol data and additionally adjusted for smoking and alcohol consumption to evaluate potential residual confounding by lifestyle factors. Fourth, we repeated the Cox regression analysis using cardiovascular mortality as the endpoint. Cardiovascular mortality was identified using the recoded leading underlying cause variable UCOD_LEADING: category 1 for all heart diseases and category 5 for cerebrovascular diseases. This two-category grouping fully corresponds to the standardized ICD-10 codes I00–I09, I11, I13, I20–I51 (heart disease) and I60–I69 (cerebrovascular disease) recommended by NCHS.

Statistical significance was determined by a two-sided *p*-value < 0.05. The analyses were carried out with R version 4.5.0, with key packages including survey, rms, survival, and mediation.

## Results

### Baseline characteristics

The study included 13,161 participants (6,531 men and 6,630 women), with the sample being weighted to reflect a population of 54,616,985 individuals. As outlined in Table 1, baseline characteristics were compared between those reporting chest pain and those without. Roughly 28% (3,725 participants) experienced chest pain, with this group tending to be older, less educated, and having a lower family PIR. Furthermore, individuals with chest pain showed higher average HbA1C, fasting glucose, BMI, WBC, waist circumstance, and an increased prevalence of hypertension, CVD, and diabetes.

**Table 1:** Baseline characteristics of participants with or without chest pain

Variables	Total (*n* = 13161)	Pain(*n* = 3725)	No pain(*n* = 9436)	*P*-value
Demographics				
Age, years	55 (47, 65)	56 (47, 66)	55 (47, 65)	0.028
Gender, (%)				0.656
Male	6531 (48.3)	1780 (47.9)	4751 (48.5)	
Female	6630 (51.7)	1945 (52.1)	4685 (51.5)	
Ethnicity, (%)				0.190
Mexican American	2098 (5.6)	557 (5.1)	1541 (5.9)	
Non-Hispanic White	6392 (75.2)	1918 (75.4)	4474 (75.1)	
Non-Hispanic Black	2546 (9.1)	744 (9.6)	1802 (8.8)	
Others	2125 (10.1)	506 (9.9)	1619 (10.2)	
Education level, (%)				< 0.001
Less than high school	3713 (17.3)	1138 (21.0)	2575 (15.8)	
High school or equivalent	2985 (23.9)	857 (25.2)	2128 (23.4)	
College or above	6463 (58.8)	1730 (53.7)	4733 (60.8)	
Family PIR	3.37 (1.75, 5.00)	2.95 (1.46, 5.00)	3.53 (1.87, 5.00)	< 0.001
Physical examination				
BMI, kg/m^2^	28.02 (24.66, 32.26)	28.64 (25.00, 33.18)	27.85 (24.51, 31.80)	< 0.001
Waist circumstance, cm	99.50 (90.00, 109.90)	101.40 (91.69, 112.04)	98.70 (89.50, 109.00)	< 0.001
SBP, mmHg	122.67 (112.67, 134.67)	122.00 (113.33, 135.33)	123.33 (112.67, 134.67)	0.459
DBP, mmHg	72.00 (64.67,78.67)	71.33 (64.00, 78.67)	72.00 (64.67, 78.67)	0.288
Medical conditions				
Hypertension, n (%)	7013 (47.6)	2300 (56.0)	4713 (44.4)	< 0.001
Diabetes, n (%)	3099 (17.9)	986 (19.6)	2113 (17.2)	0.026
Heart failure, n (%)	572 (3.4)	377 (7.9)	195 (1.6)	< 0.001
CHD, n (%)	794 (5.3)	511 (12.0)	283 (2.7)	< 0.001
Angina, n (%)	524 (3.6)	414 (10.1)	110 (1.1)	< 0.001
Heart attack, n (%)	794 (4.9)	542 (12.2)	252 (2.1)	< 0.001
Stroke, n (%)	671 (3.9)	302 (6.5)	369 (2.9)	< 0.001
Laboratory measurements				
White blood cells, 10^9^/L	6.40 (5.40, 7.80)	6.60 (5.60, 8.00)	6.30 (5.30, 7.60)	< 0.001
eGDR, mg/kg/min	7.85 (5.44, 9.66)	6.77 (4.94, 9.18)	8.24 (5.69, 9.79)	< 0.001
Fasting glucose, mmol/L	5.63 (5.22, 6.22)	5.66 (5.22, 6.25)	5.61 (5.22, 6.16)	0.044
HbA1c, %	5.50 (5.30, 5.80)	5.50 (5.30, 5.90)	5.50 (5.30, 5.80)	0.010
Total cholesterol (mmol/L)	5.15 (4.50, 5.87)	5.07 (4.42, 5.84)	5.20 (4.53, 5.87)	0.003

*Abbreviations*: *HbA1c* Hemoglobin A1c, *PIR* Poverty-to-income ratio, *eGDR* Estimated glucose disposal rate, *CHD* Coronary heart disease, *SBP* Systolic blood pressure, *DBP* diastolic blood pressure, *BMI* Body mass index

Conversely, chest pain group displayed lower levels of eGDR and TC (*p* < 0.05).

### Association between eGDR with chest pain

**Fig. 2:**
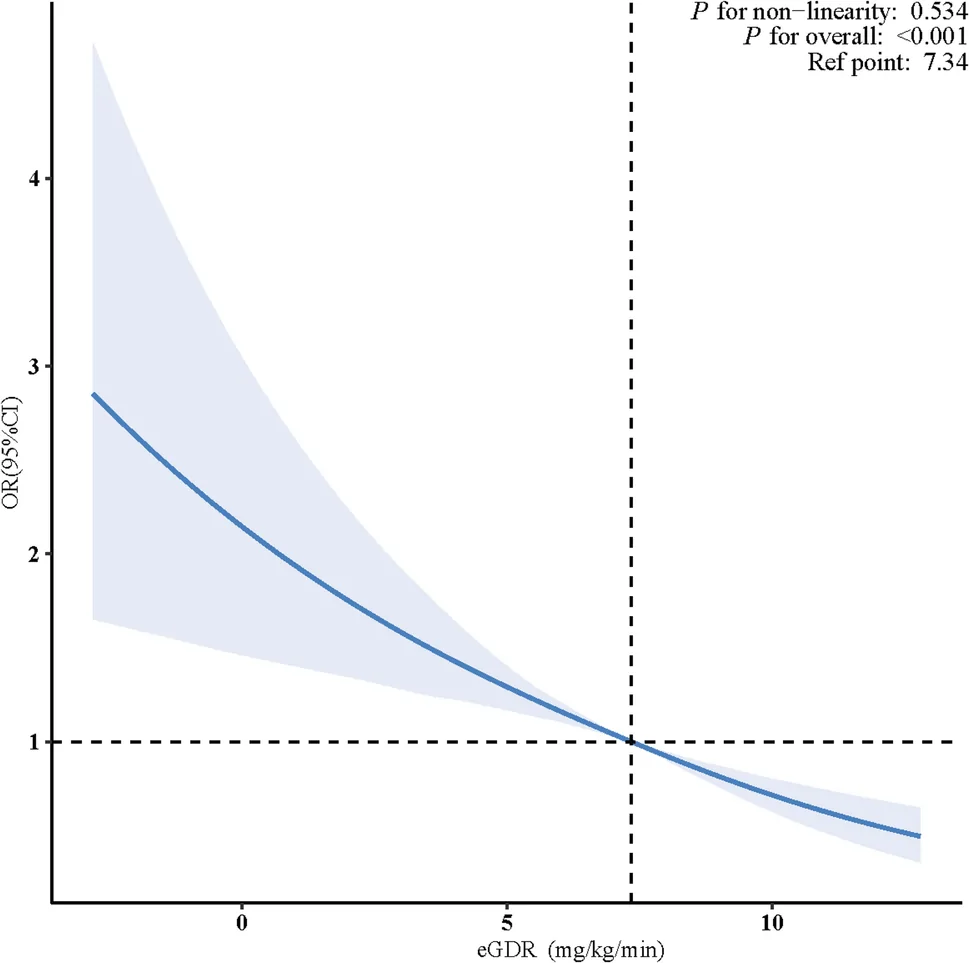
Dose-response relationship between eGDR and the prevalence of chest pain

Weighted logistic regression analysis demonstrated a robust inverse correlation between eGDR and chest pain prevalence. When examining eGDR as a continuous variable, each SD increase correlated with an 11% decrease in chest pain prevalence (OR = 0.89, 95% CI=0.86–0.92, p < 0.001, Table 2). RCS results illustrated the dose-response pattern between eGDR and the prevalence of chest pain, revealing a consistent linear inverse association across all subjects (*P* for nonlinearity = 0.534, Fig. 2). When eGDR was dichotomized at the population median (7.34 mg/kg/min), participants with high eGDR was significantly associated with decreased odds of chest pain compared with those with low eGDR in the multivariable-adjusted model (OR = 0.67, 95% CI: 0.59–0.77, *P* < 0.001), consistent with the continuous analysis. Participants in higher eGDR quartiles showed a markedly reduced prevalence of chest pain relative to the baseline group (Q1) after comprehensive adjustment for covariates. To be more specific, the ORs for chest pain prevalence were 0.77 (95% CI=0.64–0.93) in Q2, 0.61 (95% CI=0.51–0.74) in Q3, and 0.48 (95% CI=0.39–0.59) in Q4, with all *p*-value < 0.05 and a *p* for trend < 0.001 (see Supplementary Table 1 for details).

**Table 2:** Associations between eGDR and the prevalence of chest pain

Chest pain	Model 1 OR (95% CI)	*P*-value	Model 2 OR (95% CI)	*P*-value	Model 3 OR (95% CI)	*P*-value
eGDR continues	0.90(0.88,0.92)	< 0.001	0.90(0.88,0.91)	< 0.001	0.89(0.86,0.92)	< 0.001
eGDR groups						
Low eGDR (< 7.34 mg/kg/min)	Reference		Reference		Reference	
High eGDR (≥ 7.34 mg/kg/min)	0.59(0.53,0.66)	< 0.001	0.59(0.52,0.66)	< 0.001	0.67(0.59,0.77)	< 0.001

*Abbreviations:* *OR* Odd Ratio, *95%CI* 95% Confidence Interval

Model 1: Unadjusted

Model 2: Race, sex, age were adjusted

Model 3: Race, sex, age, BMI, family PIR, education level, diabetes, WBC, total cholesterol, stroke, heart attack, heart failure, angina, and coronary heart disease were adjusted

**Fig. 3:**
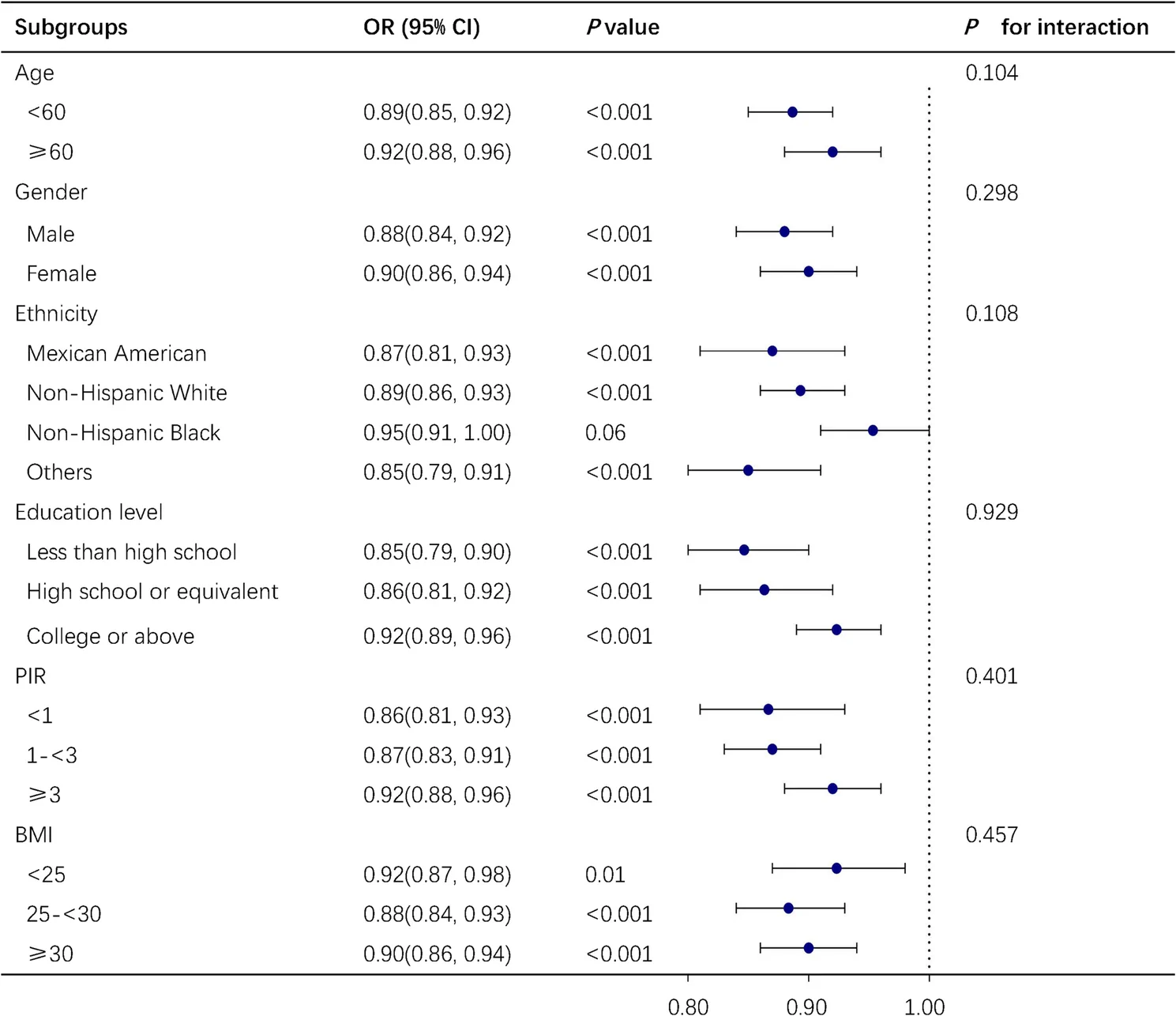
Subgroup analysis of the relationship between eGDR and chest pain prevalence. Adjusted for race, sex, age, BMI, PIR, education level, diabetes, WBC, TC, stroke, heart attack, angina, coronary heart disease, and heart failure. Abbreviations: PIR: poverty-to-income ratio, BMI: body mass index, OR: odds ratio, CI: confidence interval

To further study the correlation between eGDR and chest pain across different populations stratified by race, gender, age, BMI, PIR, and education level, subgroup analyses were conducted (Fig. 3). No significant subgroup interactions were observed (all *P* for interaction > 0.05), suggesting that the factors mentioned above do not significantly impact the observed relationship between eGDR and chest pain prevalence. 

### Correlation between eGDR and all-cause mortality

**Fig. 4:**
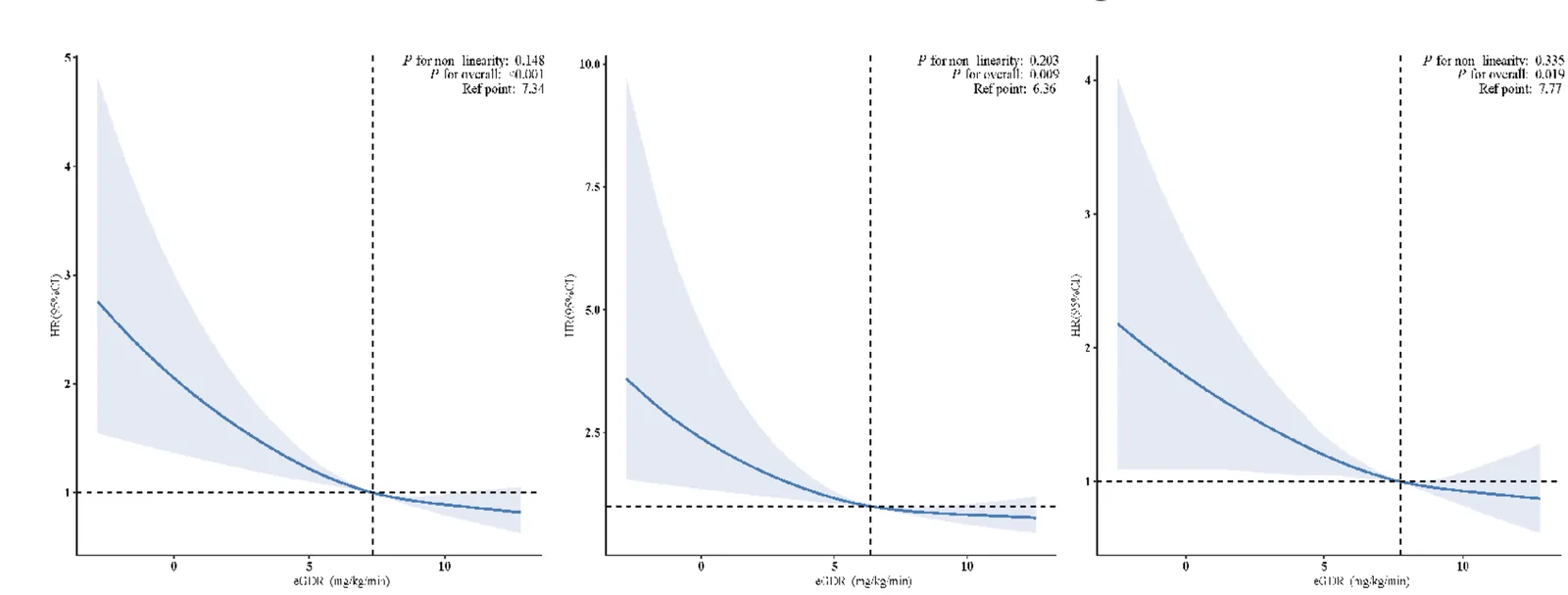
Restricted cubic spline curves for the relationship between eGDR and all-cause mortality in (**A**) total participants, (**B**) participants with chest pain, and (**C**) participants without chest pain

Over a median follow-up period of 10.42 years, 2,653 participants (20.16% of the total cohort) experienced mortality. Weighted multivariable Cox regression analysis showed that for each one-unit increase in eGDR, the HRs for mortality were 0.90 (95% CI=0.88–0.92) for the total participants, 0.93 (95% CI=0.88–0.98) for the chest pain population and 0.95 (95% CI=0.91–0.98) for the population without chest pain (Table 3). RCS analysis illustrated a linear dose-response relationship between eGDR levels and mortality risk (*p* for nonlinearity > 0.05; Fig. 4). Crucially, as eGDR values dropped, mortality risk climbed, a pattern observed uniformly across all participants, irrespective of chest pain status. We used the overall population median eGDR (7.34 mg/kg/min) as a unified cut-off to stratify participants, enabling consistent comparison across the total sample and subgroups with or without chest pain. In both the overall population and the subgroup without chest pain, the binary eGDR classification consistently yielded statistically significant inverse associations across all three models (all *P* < 0.05, Table 3). By contrast, among individuals with prevalent chest pain, models adjusted for partial covariates (Model 2: age, sex, race) and full covariates (Model 3) yielded non-significant associations (*P* > 0.05). In quartile-based analyses, the point estimates for the highest versus lowest eGDR quartile were consistently in the protective direction, although the 95% confidence intervals were wide and some contrasts did not reach statistical significance (Supplementary Table 2).

**Table 3:** Associations between eGDR and risk of all-cause mortality

Characteristic	model 1			model 2			model 3		
	HR	95% CI	*P*-value	HR	95% CI	*P*-value	HR	95% CI	*P*-value
All participants									
eGDR Continous	0.89	0.87, 0.91	< 0.001	0.90	0.88, 0.91	< 0.001	0.90	0.88, 0.92	< 0.001
eGDR groups									
Low eGDR (< 7.34 mg/kg/min)	Reference			Reference			Reference		
High eGDR (≥ 7.34 mg/kg/min)	0.55	0.50, 0.61	< 0.001	0.79	0.72, 0.87	< 0.001	0.85	0.75, 0.95	0.006
Chest Pain									
eGDR Continous	0.91	0.88, 0.93	< 0.001	0.95	0.92, 0.99	0.007	0.93	0.88, 0.98	0.004
eGDR groups									
Low eGDR (< 7.34 mg/kg/min)	Reference			Reference			Reference		
High eGDR (≥ 7.34 mg/kg/min)	0.63	0.53, 0.75	< 0.001	0.86	0.72, 1.03	0.102	0.87	0.71, 1.06	0.169
No Chest pain									
eGDR Continous	0.87	0.86, 0.89	< 0.001	0.94	0.92, 0.96	< 0.001	0.95	0.91, 0.98	0.006
eGDR groups									
Low eGDR (< 7.34 mg/kg/min)	Reference			Reference			Reference		
High eGDR (≥ 7.34 mg/kg/min)	0.53	0.47, 0.59	< 0.001	0.79	0.70, 0.88	< 0.001	0.86	0.73, 1.00	0.049

*Abbreviations: HR* Hazard ratio, *95%CI* 95% Confidence Interval

Model 1: Unadjusted

Model 2: Race, sex, age were adjusted

Model 3: Race, sex, age, BMI, family PIR, education level, diabetes, WBC, total cholesterol, stroke, heart attack, heart failure, angina, and coronary heart disease were adjusted

**Fig. 5:**
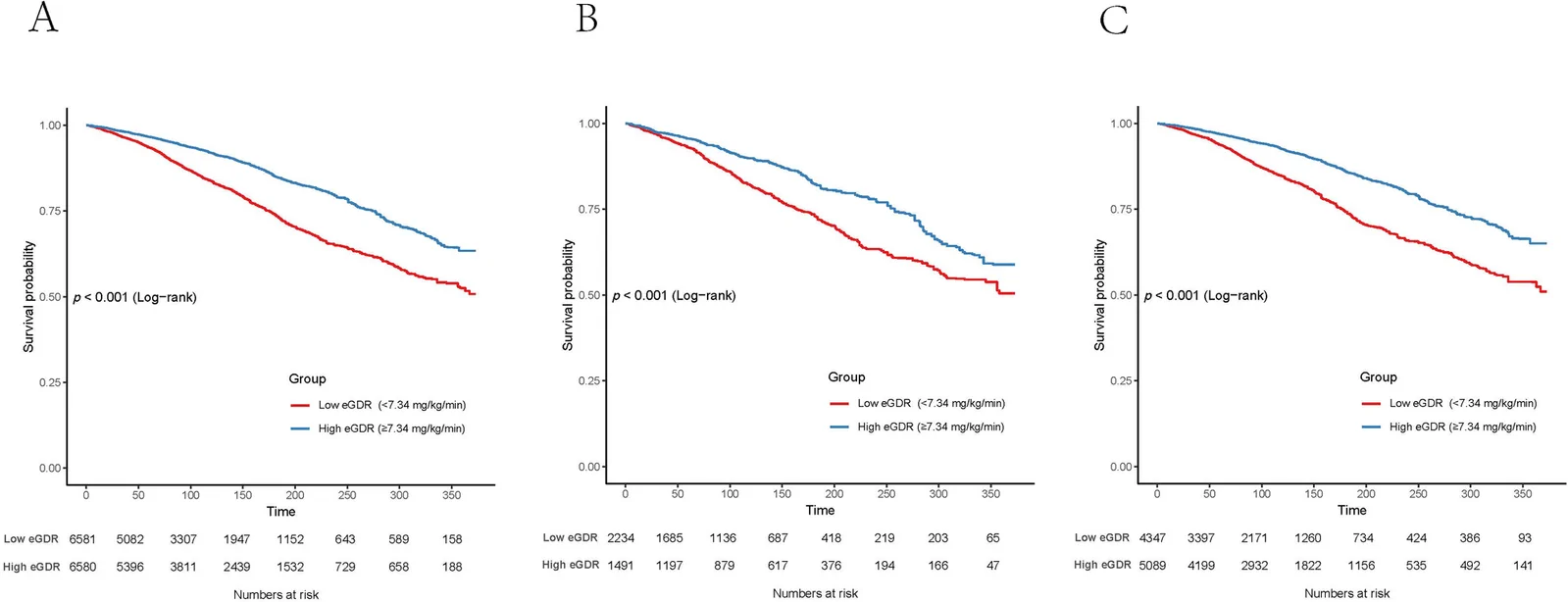
Kaplan–Meier survival curves showing all-cause mortality stratified by eGDR groups (low vs. high, cut‑off: 7.34 mg/kg/min) in (**A**) total participants, (**B**) participants with chest pain, and (**C**) participants without chest pain

Kaplan-Meier survival curves revealed that participants in the high eGDR group exhibited significantly superior overall survival in comparison with people in the low eGDR group, irrespective of the presence of chest pain (All log-rank *p* value < 0.001) (Fig. 5A, B, C). Quartile-based analyses similarly showed a graded improvement in survival from the lowest to the highest eGDR quartile (all log-rank *P* < 0.001; Supplementary Fig. S1).

**Fig. 6:**
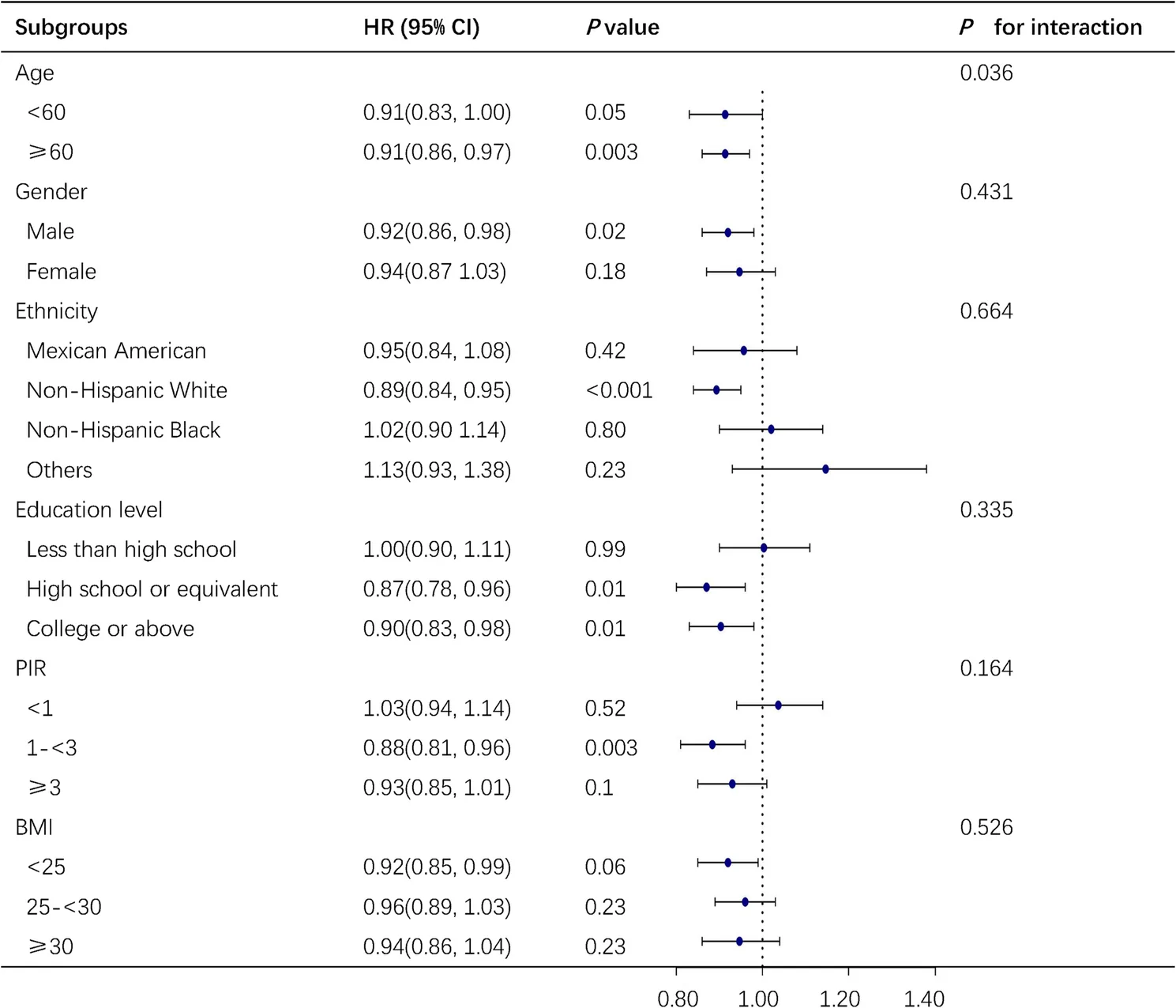
Subgroup analysis of the link between eGDR and all-cause mortality in participants with chest pain. Adjusted for race, sex, age, BMI, education level, PIR, diabetes, WBC, total cholesterol, stroke, heart attack, angina, coronary heart disease and heart failure. Abbreviations: BMI: body mass index, PIR: poverty-to-income ratio, OR: odds ratio, CI: confidence interval

**Fig. 7:**
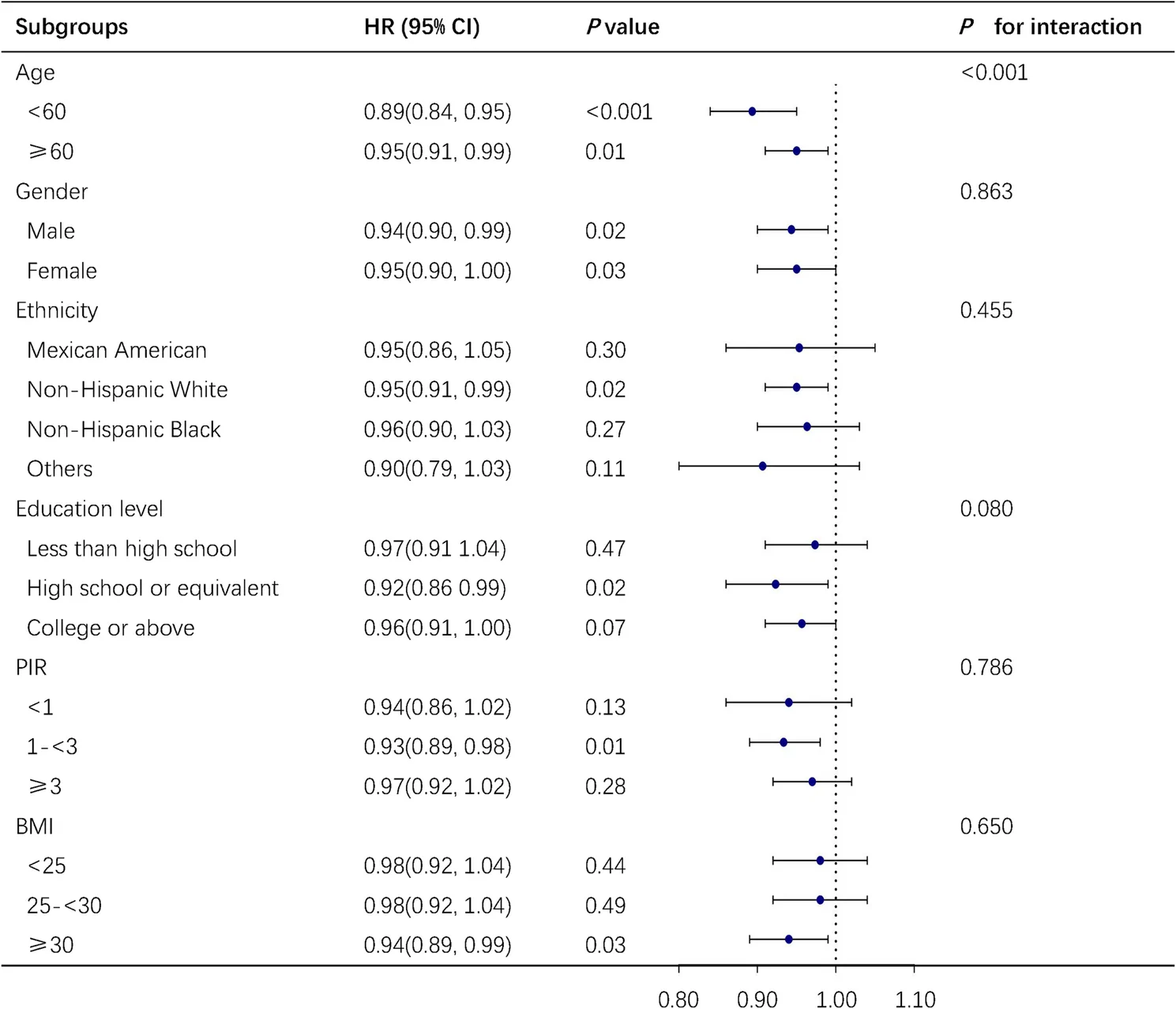
Subgroup analysis of the link between eGDR and all-cause mortality in participants without chest pain. Adjusted for race, sex, age, BMI, education level, PIR, diabetes, WBC, total cholesterol, stroke, heart attack, angina, coronary heart disease and heart failure. Abbreviations: BMI: body mass index, PIR: poverty-to-income ratio, OR: odds ratio, CI: confidence interval

Although point estimates varied in magnitude across certain strata, most notably among ethnic subgroups, where the association was nominally significant in Non-Hispanic White participants but not in other groups, formal interaction tests revealed that the link between eGDR and all-cause mortality was not modified by race, gender, education levels, BMI, and PIR in both participants with and without chest pain (all *p* for interaction > 0.05) (Fig. 6 and 7). Age was the only variable showing significant effect modification. In participants with chest pain, each 1-unit increase in eGDR correlated with a borderline reduction in mortality risk among individuals aged <60 years (adjusted HR = 0.91, 95% CI=0.83–1.00; *P* = 0.05). In contrast, a more statistically significant protective effect was observed in those aged ≥60 years (adjusted HR = 0.91, 95% CI=0.86–0.97; *P* = 0.003 ; *P* for interaction= 0.036). Similarly, in those without chest pain, the intervention showed a strong protective impact among patients under 60 years of age (adjusted HR=0.89, 95% CI=0.84-0.95, *P* < 0.001), resulting in an 11% decrease in hazard risk. However, in patients aged ≥60 years, the protective association was diminished (adjusted HR=0.95, 95% CI=0.91-0.99, *P* = 0.01).

### Mediation analyses

**Fig. 8:**
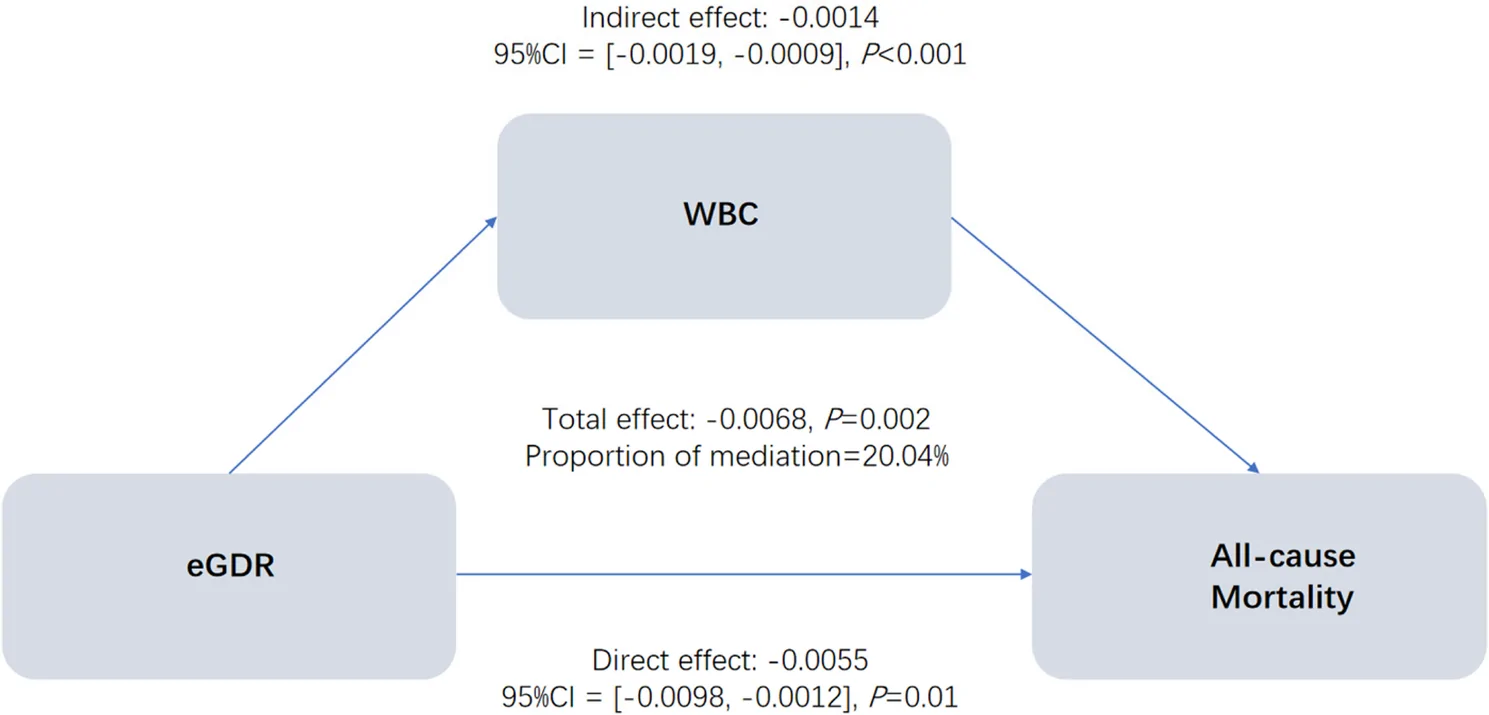
Mediation effect of WBC in the relationship between eGDR and all-cause mortality. Abbreviations: WBC, white blood cell; eGDR: estimated glucose disposal rate

To clarify the mechanisms behind the link between eGDR and survival outcomes, we did a mediation analysis with WBC as a potential mediator. Notably, mediation analysis demonstrated that 20.04% of the association between eGDR and all-cause mortality was mediated by WBC (Fig. 8). The direct effect of eGDR on all-cause mortality remained significant after controlling for WBC (Direct Effect: β = -0.0055, 95% CI=-0.0098 – -0.0012, *p* = 0.01), indicating partial mediation.

### Sensitivity analyses

The robustness of our findings was evaluated in four sensitivity analyses: (1) excluding participants with baseline cardiovascular disease; (2) excluding deaths within the first 2 years of follow-up; (3) restricting to participants with complete lifestyle data with additional adjustment for smoking and alcohol consumption; (4) examining cardiovascular mortality as the outcome.

The inverse association between eGDR and prevalent chest pain remained statistically significant across all applicable sensitivity analyses (all *P* < 0.05; Supplementary Table S3). For all-cause mortality, the protective association persisted in the overall population across all scenarios (all *P* < 0.05). In subgroup analyses, the association remained significant in virtually all scenarios, with the only exception being the chest-pain subgroup in the lifestyle-restricted analysis (N=1,390), where the estimate was attenuated and non-significant. In the corresponding non-chest-pain subgroup, in which the sample size remained adequate (N=3,353), the association was unchanged (Model 3 HR=0.92, 95% CI 0.87–0.98, *P* = 0.006, Supplementary Table S4).

For cardiovascular mortality, point estimates were consistently in the protective direction, and the Kaplan–Meier curves showed clear separation between low and high eGDR groups (all log-rank *P* < 0.05; Supplementary Figure S2 A, B, C), although statistical significance was not attained in all fully adjusted models. These findings are directionally consistent with the all-cause mortality results and provide supportive, though not conclusive, evidence for a cardiometabolic protective role of higher eGDR.

Taken together, these sensitivity analyses provide converging evidence that the observed associations of eGDR with chest pain and all-cause mortality are robust and unlikely to be substantially confounded by pre-existing cardiovascular disease, reverse causation, or lifestyle factors. 

## Discussion

### Main findings

To our knowledge, this is the first population-based study to demonstrate that eGDR is associated with both chest pain prevalence and all-cause mortality. We found a linear inverse association between eGDR and chest pain, with lower eGDR associated with a higher likelihood of chest pain. Notably, the inverse association between eGDR and all-cause mortality was observed in both participants with and without chest pain. 

Mediation analysis identified white blood cell count as a significant mediator of the eGDR-mortality association. These findings suggest that eGDR may be useful for identifying high risk individuals before symptom onset, potentially enabling early lifestyle or pharmacological interventions. 

### Comparison with previous studies 

A population based study of 10,895 adults in southeastern China reported that each standard deviation increment in eGDR was associated with a 25.9% lower risk of ischemic heart disease, underscoring the potential value of eGDR for cardiovascular risk identification [31]. Subsequent work pooling two large prospective cohorts from Europe and Asia (N = 16,656) with repeated eGDR measurements demonstrated that individuals with persistently low or declining eGDR had an elevated risk of incident cardiovascular disease [32]. Although chest pain is the most common symptomatic manifestation of cardiovascular disease, the association between eGDR and chest pain has rarely been examined. In the present study, we observed a consistent inverse association between eGDR and chest pain prevalence that persisted after extensive multivariable adjustment and was supported by subgroup analyses.

A growing body of evidence has linked eGDR to mortality risk across diverse populations. Giuseppe and his team, in a follow-up investigation of 15,773 individuals with type 2 diabetes, identified that eGDR was connected to the development of CVD and the risk of long-term mortality [33]. More recently, Xiaohan Ma's work reported a strong inverse correlation between eGDR and mortality risk in patients with the cardiovascular-kidney-metabolic syndrome, with higher eGDR corresponding to significantly lower all-cause and cardio-cerebrovascular mortality [34]. Consistent with these observations, our findings demonstrate an inverse association of eGDR with all-cause mortality among individuals with chest pain, and a similarly protective association in those without chest pain. This association remained robust across all adjusted models and across prespecified subgroups. 

### Potential mechanisms

It is hypothesized that eGDR, when considered as an uncomplicated indicator of IR, may be linked to chest pain and mortality via the following mechanisms. In the IR condition, β-cells perceive the intensity of IR and elevate insulin secretion to offset the inadequate insulin action [35]. Numerous investigations have demonstrated that elevated insulin levels have the capacity to speed up the atherosclerotic process via different mechanisms.

These include: (1) promoting the growth of smooth muscle cells, the cross-linking of collagen, and the accumulation of collagen [36], (2) worsening inflammation and oxidative stress [37], (3) leading to excessive platelet activity and abnormal adhesion initiation [38], and (4) facilitating the movement of low-density lipoprotein cholesterol into arterial smooth muscle cells [39]. The combined impact of pathological alterations can lead to problems like endothelial dysfunction and greater vascular stiffness, which may eventually cause structural and functional heart abnormalities [40, 41]. To manage the eGDR index effectively, we consider interventions like regulating blood sugar and waist size, alongside preventive strategies such as enhancing diet and boosting physical activity. In the future, we plan to explore the molecular mechanisms that explain how the eGDR index affects chest pain and mortality.

Additionally, we carried out a mediation analysis to understand the role of WBC in the connection between eGDR and overall mortality. We discovered a substantial indirect influence of eGDR on all-cause mortality, with WBC as a mediator, implying that eGDR might raise mortality rates by affecting inflammation. According to a prior study, there is a significant relationship between WBC and insulin resistance assessed using eGDR (r=-0.14, *P* ≤ 0.01) [42]. The mechanism behind the joint effect of these markers is as follows: excessive visceral fat buildup in obesity can trigger localized tissue ischemia and hypoxia, creating conditions that promote fat cell breakdown and attract immune cells like macrophages [43]. Inflammatory mediators, particularly interleukin-6 and tumor necrosis factor-alpha, are activated by this process. These mediators contribute to the worsening of IR by accelerating lipolysis in adipocytes and elevating serine/threonine phosphorylation levels of insulin receptor substrate-1 (IRS-1) [44]. In comparison to conventional IR markers such as HIEG and HOMA-IR, eGDR is simpler to acquire and less expensive. In contrast to the Triglyceride-glucose (TyG) index, eGDR combines lab results with clinical data, providing a more thorough representation of insulin resistance. 

### Strengths and limitations

Our study’s key strength lies in its robust findings, which are based on a substantial and demographically diverse sample of the U.S. population, significantly bolstering the research’s credibility. The application of sampling weights further strengthens the statistical precision and dependability of our conclusions. Nonetheless, several limitations must be acknowledged. First, despite comprehensive covariate adjustment, residual or unmeasured confounding may persist. Four lifestyle covariates (smoking, alcohol consumption, physical activity, and depressive symptoms) were not included in the primary regression models. Complete smoking and alcohol records were limited and would have led to substantial sample loss and selection bias. Physical activity and mental health questionnaires employed different assessment tools across survey cycles, making harmonization infeasible. A sensitivity analysis restricted to participants with complete smoking and alcohol data yielded results consistent with the primary findings. However, residual confounding by unadjusted lifestyle factors remains a limitation. Second, the cross-sectional assessment of chest pain precludes causal inference regarding its association with eGDR. Third, owing to the nature of the NHANES database, we could not examine the dynamic, longitudinal relationship between eGDR and health outcomes.

Fourth, because our analysis relied exclusively on U.S. data, the findings may not be directly generalizable to other populations. Future multi-center studies are warranted to assess external validity. Fifth, self-reported chest pain and lifestyle data are subject to recall bias and potential outcome misclassification. Sixth, although subgroup analyses were pre-specified and evaluated using interaction tests, no formal correction for multiple comparisons was applied because the primary aim was to assess the consistency of associations across strata rather than to test multiple independent hypotheses. This approach increases the likelihood of false-positive findings, particularly in smaller subgroups. Accordingly, subgroup results should be interpreted as exploratory. Notably, although point estimates varied in magnitude across certain strata, most notably among racial subgroups, the non-significant interaction tests indicate that these differences are likely driven by unequal sample sizes and statistical power rather than true biological heterogeneity. We therefore caution against interpreting subgroup-specific P-values in isolation. Seventh, our mediation analysis relied solely on baseline WBC count, a crude and non-specific inflammatory marker that is susceptible to transient elevations from acute infection or stress. Single-time-point measurement further introduces misclassification of sustained inflammatory status. The modest mediated proportion (20.04%), although statistically significant, suggests that leukocyte-related inflammation only partially accounts for the eGDR-mortality association. Serum CRP was not evaluated as an alternative mediator because CRP laboratory data were unavailable for the 2011-2012 and 2013-2014 NHANES cycles, which would have led to severe sample attrition. Moreover, assay methods were inconsistent across cycles, with conventional CRP used from 1999-2010 and high-sensitivity CRP adopted after 2015. Future studies incorporating repeated, specific inflammatory biomarkers and Bayesian mediation approaches are warranted to validate our findings. Eighth, cardiovascular mortality was not pre-specified as a secondary endpoint, although it was examined in a sensitivity analysis and yielded directionally consistent results. Dedicated studies with pre-specified cardiovascular mortality endpoints are needed to confirm the cardiometabolic specificity of the eGDR-mortality association. Finally, we note that both the binary eGDR classification (based on the overall population median of 7.34 mg/kg/min) and the quartile-based categorization showed reduced statistical significance in the chest-pain subgroup after full adjustment. This is partially attributable to the lower median eGDR in this subgroup (6.36 mg/kg/min), which, when the population-level cut-off was applied, resulted in a sparsely populated high-eGDR stratum and limited statistical power. The quartile analysis further compounded this issue through the inherent loss of information when discretizing a continuous variable. We therefore emphasize that the continuous and RCS-based analyses, which use the full information in the data, are more reliable and should be prioritized over categorical estimates. The quartile results are retained in the Supplementary Material for completeness but should be interpreted with appropriate caution. 

## Conclusion

This nationally representative study demonstrated that a higher eGDR was robustly and inversely associated with both chest pain prevalence and long-term all-cause mortality, irrespective of chest pain status. The partial mediation of this mortality association by white blood cells suggested a plausible inflammatory pathway. These findings support the potential utility of eGDR as a non-invasive marker for cardiometabolic risk stratification.

Further longitudinal studies in diverse populations are warranted to validate these results and to fully evaluate the potential of eGDR integration into routine clinical practice.

## Supplementary Information

Supplementary Material 1.

## Data Availability

Data can be found online (https://wwwn.cdc.gov/nchs/nhanes/default.aspx).
