# Supplementary material for: Associations of estimated glucose disposal rate with chest pain prevalence and mortality risks: a population-based study
Source: BMC Cardiovasc Disord. 2026 Jul 25;26:954. doi: 10.1186/s12872-026-06336-0 (PMC13685353; doi:10.1186/s12872-026-06336-0)
Supplement: Supplementary file 1 [file 12872_2026_6336_MOESM1_ESM.docx]

**Associations of estimated glucose disposal rate with chest pain prevalence and mortality risks: a population-based study**

Jieke Wang ^1^, Manli Ye ^2*^

**Supplementary Material**

Table S1. Associations between eGDR and the prevalence of chest pain

Table S2. Associations between quartile-stratified eGDR and risk of all-cause mortality

Table S3. Sensitivity analyses of the associations between eGDR with chest pain, all-cause mortality, and cardiovascular mortality.

Table S4. Sensitivity analyses of the associations between eGDR with all-cause mortality and cardiovascular mortality, stratified by chest pain status

Figure S1. Kaplan–Meier survival curves showing all-cause mortality stratified by eGDR quartiles levels in (A) total participants, (B) participants with chest pain, and (C) participants without chest pain.

Figure S2. Kaplan–Meier survival curves showing cardiovascular mortality stratified by eGDR group (low vs. high, cut‑off: 7.34 mg/kg/min) in (**A**) total participants, (**B**) participants with chest pain, and (**C**) participants without chest pain.

**Table S1.** Associations between eGDR and the prevalence of chest pain

| **Chest pain** | **Model 1  OR (95% CI)** | ***P*-value** | **Model 2  OR (95% CI)** | ***P*-value** | **Model 3  OR (95% CI)** | ***P*-value** |
| --- | --- | --- | --- | --- | --- | --- |
| eGDR quartiles |  |  |  |  |  |  |
| Q1 | Reference |  | Reference |  | Reference |  |
| Q2 | 0.81(0.70,0.92) | 0.002 | 0.80(0.70,0.92) | 0.002 | 0.77(0.64,0.93) | 0.008 |
| Q3 | 0.59(0.50,0.68) | <0.001 | 0.58(0.50,0.68) | <0.001 | 0.61(0.51,0.74) | <0.001 |
| Q4 | 0.48(0.43,0.55) | <0.001 | 0.47(0.41,0.54) | <0.001 | 0.48(0.39,0.59) | <0.001 |
| *P* for trend |  | <0.001 |  | <0.001 |  | <0.001 |

Abbreviations: OR, Odd Ratio; 95%CI, 95% Confidence Interval

Model 1: Unadjusted

Model 2: Race, sex, age were adjusted

Model 3: Race, sex, age, BMI, family PIR, education level, diabetes, WBC, total cholesterol, stroke, heart attack, heart failure, angina, and coronary heart disease were adjusted.

**Table S2.** Associations between quartile-stratified eGDR and risk of all-cause mortality

| **Characteristic** | **model 1** |  |  | **model 2** |  |  | **model 3** |  |  |
| --- | --- | --- | --- | --- | --- | --- | --- | --- | --- |
|  | **HR** | **95% CI** | ***P*-value** | **HR** | **95% CI** | ***P*-value** | **HR** | **95% CI** | ***P*-value** |
| **All participants** |  |  |  |  |  |  |  |  |  |
| eGDR |  |  |  |  |  |  |  |  |  |
| Q1 | Reference |  |  | Reference |  |  | Reference |  |  |
| Q2 | 0.89 | 0.79, 1.01 | 0.080 | 0.93 | 0.81, 1.05 | 0.245 | 0.98 | 0.85, 1.13 | 0.755 |
| Q3 | 0.61 | 0.53, 0.70 | <0.001 | 0.80 | 0.69, 0.92 | 0.002 | 0.87 | 0.74, 1.03 | 0.101 |
| Q4 | 0.44 | 0.39, 0.50 | <0.001 | 0.72 | 0.64, 0.81 | <0.001 | 0.77 | 0.64, 0.92 | 0.005 |
| *P* for trend |  |  | <0.001 |  |  | <0.001 |  |  | 0.002 |
| **Chest Pain** |  |  |  |  |  |  |  |  |  |
| eGDR |  |  |  |  |  |  |  |  |  |
| Q1 | Reference |  |  | Reference |  |  | Reference |  |  |
| Q2 | 0.88 | 0.69, 1.13 | 0.327 | 0.87 | 0.68, 1.11 | 0.250 | 0.79 | 0.58, 1.08 | 0.134 |
| Q3 | 0.76 | 0.58, 1.00 | 0.049 | 0.85 | 0.65, 1.13 | 0.261 | 0.78 | 0.56, 1.10 | 0.154 |
| Q4 | 0.52 | 0.42, 0.65 | <0.001 | 0.79 | 0.63, 1.01 | 0.056 | 0.69 | 0.47, 1.01 | 0.055 |
| *P* for trend |  |  | <0.001 |  |  | 0.083 |  |  | 0.080 |
| **No Chest pain** |  |  |  |  |  |  |  |  |  |
| eGDR |  |  |  |  |  |  |  |  |  |
| Q1 | Reference |  |  | Reference |  |  | Reference |  |  |
| Q2 | 0.78 | 0.68, 0.91 | 0.001 | 0.87 | 0.76, 1.00 | 0.051 | 0.97 | 0.83, 1.14 | 0.741 |
| Q3 | 0.52 | 0.45, 0.61 | <0.001 | 0.73 | 0.62, 0.86 | <0.001 | 0.82 | 0.68, 1.00 | 0.046 |
| Q4 | 0.42 | 0.36, 0.48 | <0.001 | 0.72 | 0.62, 0.84 | <0.001 | 0.83 | 0.66, 1.04 | 0.113 |
| *P* for trend |  |  | <0.001 |  |  | <0.001 |  |  | 0.032 |

Abbreviations: HR = Hazard Ratio, CI = Confidence Interval

Model 1: Unadjusted

Model 2: Adjusted for age, sex, and race

Model 3: Adjusted for age, gender, race, BMI, family income poverty ratio, education level, diabetes, WBC, NLR, TC, stroke, heart attack, angina, coronary heart disease and heart failure

**Table S3.** Sensitivity analyses of the associations between eGDR with chest pain, all-cause mortality, and cardiovascular mortality.

| **Analysis Type** | **Outcome** | **Valid n** | **Effect Measure** | **Model 1** | **Model 2** | **Model 3** |
| --- | --- | --- | --- | --- | --- | --- |
| Primary Analysis (Reference) | Prevalent chest pain | 13161 | OR | 0.90(0.88,0.92), *P*<0.001 | 0.90(0.88,0.91), *P*<0.001 | 0.89(0.86,0.92), *P*<0.001 |
|  | All-cause mortality | 13161 | HR | 0.89(0.87,0.91), *P*<0.001 | 0.90(0.88,0.91), *P*<0.001 | 0.90(0.88,0.92), *P*<0.001 |
|  | CVD-specific mortality | 13161 | HR | 0.97(0.95,0.98), *P*<0.001 | 0.97(0.96,0.98), *P*<0.001 | 0.98(0.97,1.00), *P*=0.027 |
| Sensitivity Analysis 1: Excluding participants with baseline CVD | Prevalent chest pain | 11165 | OR | 0.94(0.92,0.96), *P*<0.001 | 0.92(0.90,0.94), *P*<0.001 | 0.89(0.87,0.92), *P*<0.001 |
|  | All-cause mortality | 11165 | HR | 0.89(0.88,0.91), *P*<0.001 | 0.95(0.93,0.97), *P*<0.001 | 0.95(0.92,0.99), *P*=0.009 |
|  | CVD-specific mortality | 11165 | HR | 0.97(0.95,0.98), *P*<0.001 | 0.97(0.96,0.99), *P*<0.001 | 0.99(0.97,1.00), *P*=0.146 |
| Sensitivity Analysis 2: Excluding early deaths within first 2 years of follow-up | Prevalent chest pain | 12215 | OR | N/A | N/A | N/A |
|  | All-cause mortality | 12215 | HR | 0.88(0.87,0.90), *P*<0.001 | 0.94(0.92,0.96), *P*<0.001 | 0.94(0.91,0.97), *P*<0.001 |
|  | CVD-specific mortality | 12215 | HR | 0.97(0.95,0.98), *P*<0.001 | 0.97(0.96,0.98), *P*<0.001 | 0.98(0.97,1.00), *P*=0.051 |
| Sensitivity Analysis 3: with complete smoking/alcohol data, additional lifestyle adjustment | Prevalent chest pain | 4743 | OR | 0.91(0.88,0.94), *P*<0.001 | 0.91(0.88,0.94), *P*<0.001 | 0.93(0.88,0.99), *P*=0.023 |
|  | All-cause mortality | 4743 | HR | 0.92(0.89,0.95), *P*<0.001 | 0.95(0.91,0.98), *P*=0.002 | 0.94(0.89,0.99), *P*=0.024 |
|  | CVD-specific mortality | 4743 | HR | 0.99(0.97,1.00), *P*=0.086 | 0.99(0.97,1.00), *P*=0.110 | 0.97(0.97,1.01), *P*=0.188 |

All estimates are from survey-weighted regression models (logistic for chest pain, Cox proportional hazards for all-cause mortality, and cause-specific Cox for cardiovascular mortality). eGDR was analyzed as a continuous variable (per 1‑unit increase).

Abbreviations: CVD = cardiovascular disease; OR = odds ratio; HR = hazard ratio; CI = confidence interval. N/A: Not applicable. Cross-sectional chest pain outcome is not affected by follow-up time exclusion.

Model 1: Unadjusted

Model 2: Race, sex, age were adjusted

Model 3: Race, sex, age, BMI, family PIR, education level, diabetes, WBC, total cholesterol, stroke, heart attack, heart failure, angina, and coronary heart disease were adjusted. Smoking status and alcohol consumption were adjusted only in the sensitivity analysis 3. For sensitivity analyses excluding participants with baseline cardiovascular disease (coronary heart disease, angina, myocardial infarction, heart failure, stroke), these five CVD-related covariates were removed from Model 3 adjustment.

**Table S4.** Sensitivity analyses of the associations between eGDR with all-cause mortality and cardiovascular mortality, stratified by chest pain status

| **Subgroup** | **Analysis Type** | **Outcome** | **Valid n** | **Effect Measure** | **Model 1** | **Model 2** | **Model 3** |
| --- | --- | --- | --- | --- | --- | --- | --- |
| With chest pain | Primary Analysis (Reference) | All-cause mortality | 3725 | HR | 0.91(0.88-0.93), *P*<0.001 | 0.95(0.92-0.99), *P*=0.007 | 0.93(0.88-0.98), *P*=0.004 |
|  |  | CVD-specific mortality | 3725 | HR | 0.97(0.95-0.99), *P*<0.001 | 0.98(0.96-0.99), *P*=0.011 | 0.98(0.95-1.00), *P*=0.071 |
|  | Sensitivity Analysis 1: Excluding participants with baseline CVD | All-cause mortality | 2591 | HR | 0.92(0.89-0.96), *P*<0.001 | 0.97(0.93-1.01), *P*=0.162 | 0.93(0.86-1.00), *P*=0.067 |
|  |  | CVD-specific mortality | 2591 | HR | 0.98(0.96-1.00), *P*=0.033 | 0.99(0.97-1.01), *P*=0.199 | 0.99(0.96-1.02), *P=*0.367 |
|  | Sensitivity Analysis 2: Excluding early deaths within first 2 years of follow-up | All-cause mortality | 3397 | HR | 0.90(0.88-0.93), *P*<0.001 | 0.95(0.92-0.99), *P*=0.006 | 0.92(0.87-0.97), *P*=0.002 |
|  |  | CVD-specific mortality | 3397 | HR | 0.97(0.95-0.99), *P*<0.001 | 0.97(0.96-0.99), *P*=0.008 | 0.97(0.95-1.00), *P*=0.048 |
|  | Sensitivity Analysis 3: with complete smoking/alcohol data, additional lifestyle adjustment | All-cause mortality | 1390 | HR | 0.97(0.91-1.03), *P*=0.350 | 1.01(0.94-1.10), *P*=0.738 | 1.03(0.93-1.15), *P*=0.535 |
|  |  | CVD-specific mortality | 1390 | HR | 1.04(0.97-1.13), *P*=0.276 | 0.99(0.89-1.11), *P*=0.912 | 0.95(0.83-1.09), *P*=0.479 |
| Without chest pain | Primary Analysis (Reference) | All-cause mortality | 9436 | HR | 0.87(0.86-0.89), *P*<0.001 | 0.94(0.92-0.96), *P*<0.001 | 0.95(0.91-0.98), *P*=0.006 |
|  |  | CVD-specific mortality | 9436 | HR | 0.96(0.95-0.97), *P*<0.001 | 0.97(0.95-0.98), *P*<0.001 | 0.98(0.97-1.00), *P=*0.070 |
|  | Sensitivity Analysis 1: Excluding participants with baseline CVD | All-cause mortality | 8574 | HR | 0.88(0.86-0.90), *P*<0.001 | 0.95(0.92-0.97), *P*<0.001 | 0.96(0.92-1.00), *P*=0.057 |
|  |  | CVD-specific mortality | 8574 | HR | 0.96(0.95-0.98), *P*<0.001 | 0.97(0.95-0.98), *P*<0.001 | 0.99(0.97-1.00), *P*=0.112 |
|  | Sensitivity Analysis 2: Excluding early deaths within first 2 years of follow-up | All-cause mortality | 8818 | HR | 0.97(0.95-0.98), *P*<0.001 | 0.94(0.91-0.96), *P*<0.001 | 0.95(0.91-0.99), *P*=0.013 |
|  |  | CVD-specific mortality | 8818 | HR | 0.96(0.95-0.98), *P*<0.001 | 0.97(0.95-0.98), *P*<0.001 | 0.99(0.97-1.00), *P*=0.123 |
|  | Sensitivity Analysis 3: with complete smoking/alcohol data, additional lifestyle adjustment | All-cause mortality | 3353 | HR | 0.91(0.87-0.95), *P*<0.001 | 0.93(0.89-0.97), *P*<0.001 | 0.92(0.87-0.98), *P*=0.006 |
|  |  | CVD-specific mortality | 3353 | HR | 0.98(0.97-1.00), *P*=0.049 | 0.98(0.97-1.00), *P*=0.051 | 0.99(0.97-1.02), *P*=0.442 |

All estimates are from survey-weighted regression models (Cox proportional hazards for all-cause mortality, and cause-specific Cox for cardiovascular mortality). eGDR was analyzed as a continuous variable (per 1‑unit increase).

Abbreviations: CVD = cardiovascular disease; HR = hazard ratio; CI = confidence interval.

Model 1: Unadjusted

Model 2: Race, sex, age were adjusted

Model 3: Race, sex, age, BMI, family PIR, education level, diabetes, WBC, total cholesterol, stroke, heart attack, heart failure, angina, and coronary heart disease were adjusted. Smoking status and alcohol consumption were adjusted only in the sensitivity analysis 3. For sensitivity analyses excluding participants with baseline cardiovascular disease (coronary heart disease, angina, myocardial infarction, heart failure, stroke), these five CVD-related covariates were removed from Model 3 adjustment.

| A   | B | C |
| --- | --- | --- |

**Figure S1.** Kaplan–Meier survival curves showing all-cause mortality stratified by eGDR quartiles in (A) total participants, (B) participants with chest pain, and (C) participants without chest pain.

| A   | B | C |
| --- | --- | --- |

**Figure S2.** Kaplan–Meier survival curves showing cardiovascular mortality stratified by eGDR group (low vs. high, cut‑off: 7.34 mg/kg/min) in (**A**) total participants, (**B**) participants with chest pain, and (**C**) participants without chest pain.
